# Analysis of influencing factors on long COVID in COVID-19 patients infected with omicron variant three months after discharge: a cross-sectional study

**DOI:** 10.1186/s12879-023-08947-w

**Published:** 2024-01-02

**Authors:** Hang Shang, Tianying Chang, Wei Yang, Li Shi, Shaodan Hu, Lin Tian, Jixiang Ren, Tan Wang, Jian Wang, Jiajuan Guo, Yingzi Cui

**Affiliations:** 1 Changchun University of Chinese Medicine, Changchun, China; 2 EBM office, the Affiliated Hospital to Changchun University of Chinese Medicine, Changchun, China; 3 Pulmonary Disease Center, the Affiliated Hospital to Changchun University of Chinese Medicine, Changchun, China; 4 the Affiliated Hospital to Changchun University of Chinese Medicine, Changchun, China

**Keywords:** COVID-19, Long COVID, Omicron variant, Cross-sectional study

## Abstract

**Background:**

The purpose of this study is to analyze the influencing factors associated with Long-COVID in patients infected with Omicron variant of COVID-19 in Changchun City, Jilin Province, China three months after discharge in March 2022.

**Methods:**

In this study, we conducted a telephone follow-up based on the real-world data collected from the Affiliated Hospital to Changchun University of Chinese Medicine, Changchun Tongyuan Shelter Hospital and Changchun Infectious Disease Hospital during the COVID-19 epidemic in Changchun in March 2022. We used the Global COVID-19 Clinical Platform Case Report Form for Post COVID condition as a follow-up questionnaire to collect the general information, past medical history, clinical symptoms, COVID-19 vaccine inoculation doses, and other relevant information to analyze the symptom characteristics of COVID-19 patients three months after discharge from the hospital and related factors affecting Long COVID.

**Results:**

A total of 1,806 patients with COVID-19 were included in this study, 977 males and 829 females, with a mean age of 38.5 [30.0, 49.4] years, and the number of female patients suffering from Long COVID (50.87%) was greater than male patients (*p* = 0.023). The binary logistic regression analysis of factors influencing Long COVID showed that smoking history (OR (95%CI) = 0.551(0.425–0.714), *p* < 0.001, taking never smoking as a reference), allergy history (OR (95%CI) = 1.618 (1.086–2.413), *p*-value 0.018, taking no allergy as a reference), first symptoms (OR (95%CI) = 0.636 (0.501–0.807), *p* < 0.001, with no first symptoms as reference) and COVID-19 vaccine inoculation doses (OR (95%CI) = 1.517 (1.190–1.933), *p*-value 0.001, with ≤ 2 doses of COVID-19 vaccine inoculation doses as reference) constituted its influencing factors. The first symptoms of patients on admission mainly included fever (512 cases, 71.81%), cough (279 cases, 39.13%) and dry or itchy throat (211 cases, 29.59%). The most common symptoms of Long COVID were persistent fatigue (68 cases), amnesia (61 cases), insomnia (50 cases) and excessive sweating (50 cases).

**Conclusion:**

The first symptoms on admission were predominantly fever, cough and dry or itchy throat. The most common symptoms of Long COVID were persistent fatigue, amnesia, insomnia and excessive sweating, and female patients were at a higher risk of Long COVID.

**Supplementary Information:**

The online version contains supplementary material available at 10.1186/s12879-023-08947-w.

## Introduction

The global outbreak of Corona Virus Disease 2019 (COVID-19) occurred in December 2019, causing worldwide concern [1]. A total of five virus variants have been identified worldwide now, and Omicron variant [2], characterized by significantly increased infectivity and transmissibility, is the main prevalent variant now, but its pathogenicity has significantly decreased, and most infected patients are asymptomatic or of mild symptoms [3]. Based on the pathogenic characteristics of the Omicron variant, the number of people recovering from COVID-19 is increasing. There is an increasing amount of observational data suggesting that patients may develop various symptoms after recovery from COVID-19, some of which may cause impaired pulmonary function and corresponding imaging changes, which are defined as long-term sequelae of COVID-19 (Long COVID) [4]. As defined by the World Health Organization in October 2021 Long COVID refers to symptoms occurring at least three months after probable or confirmed severe acute respiratory syndrome coronavirus 2 (SARS-CoV-2) infection, and the symptoms must last at least two months and cannot be explained by an alternative diagnosis. These symptoms may be new onset following recovery from SARS-CoV-2 infection or persist from the initial illness [5], seriously affecting patients’ daily life and work, threatening their health, and increasing their medical burden.

Long COVID will possibly have an impact on patients’ work and life, cause or exacerbate their mental distress, and increase their psychological burden [6]. It was found in a study that 45.2% of COVID-19 patients reduced their working hours during the follow-up period and 22.3% did not work during the survey period due to their illness [7]. Studies have also shown that the condition of COVID-19 patients deteriorates after recovery and they are also prone to long-term infection, which will have a certain impact on exercise, work and life. [8]. In addition, Long COVID may also exacerbate the impact on social inequalities in terms of gender, race, education, labor market and professional activities [9]. Long COVID seriously affect people’s daily life, increase their economic and medical burden, and cause serious psychological distress, so it will be helpful to address this distress in clinical practice to conduct a useful exploration of the factors influencing Long COVID. Based on the telephone follow-up and the collection of relevant information of COVID-19 patients after discharge from the Affiliated Hospital to Changchun University of Chinese Medicine, Changchun Tongyuan Shelter Hospital and Changchun Infectious Disease Hospital in March 2022 during the outbreak of Omicron COVID-19 variant in Changchun city, Jilin province, we clarified the symptoms and characteristics of patients infected with Omicron variant of COVID-19 three months after discharge were and analyzed the risk factors of Long COVID in this study, providing references basis and assistance for the clinical prevention and treatment of Long COVID.

## Materials and methods

### Design and participants

We conducted a telephone follow-up to ask patients about their basic information and clinical symptoms in the COVID-19 Specialized Disease Database established by the real-world data collected during the COVID-19 epidemic in Changchun City in March 2022 from the Affiliated Hospital to Changchun University of Chinese Medicine, Changchun Tongyuan Shelter Hospital and Changchun Infectious Disease Hospital. Smoking history was divided into two categories: smoking and no smoking; past medical history and allergy history were divided into two categories: presence and absence; length of hospital stay was divided into two categories: > 14 days and ≤ 14 days; readmission, namely, readmission of COVID-19 patients after discharge, was divided into two categories: readmission and no readmission; COVID-19 vaccine inoculation doses were divided into two categories: basic vaccination and booster vaccination(basic vaccination refers to inoculation doses ≤ 2, while booster vaccination refers to inoculation doses > 2). The patients were then asked a questionnaire, which was based on the Global COVID-19 Clinical Platform Case Report Form for Post COVID condition, with 12 items, each with five options, assigned a score of 0–4, and then the total scores of the patients were counted and recorded. All data collected were compiled and analyzed for symptom characteristics of COVID-19 patients three months after discharge and risk factors affecting Long COVID. The study was approved by the Ethics Committee of the Affiliated Hospital to Changchun University of Chinese Medicine (Approval No: CCZYFYLL2022 review-020; CCZYFYLL2022 review-021), and all patients gave verbal informed consent prior to participation.

**Fig. 1:**
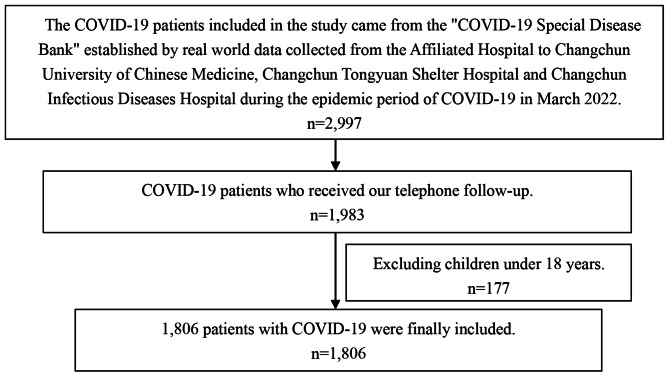
The screening flowchart for this study

In this study, 2,997 patients with COVID-19 were included, of which 1983 cases (66.17%) participated in our follow-up survey. Among the 1,983 patients, minors aged < 18 years (177 cases) were excluded, because the prevalence, presentation and characteristics of Long COVID in children are different from those in adults [10, 11].This study mainly analyzed the clinical characteristics of Long COVID in adults and the influencing factors, so 1,806 patients were finally included in this study, with the specific screening flow chart shown in Fig. 1.

### Data processing and statistical analysis

All collected follow-up questionnaires were entered into Epidata 3.1 for collation, and SPSS 25.0 was also used for statistical analysis of the data. Enumeration data were described by frequency and percentage, and χ2 test was used for intergroup comparison; measurement data conforming to normal distribution were described by mean and standard deviation, and measurement data not conforming to normal distribution were described by median [*P*_25_, *P*_75_]. Risk factors affecting Long COVID were analyzed using binary logistic regression, with the presence or absence of Long COVID as the dependent variable, age and gender as independent variables forced into the model, and the remaining statistically significant independent variables gradually entered into the model according to the forward: LR method, and *p* < 0.05 indicated the difference was statistically significant.

## Results

### Patient general information

The basic characteristics of the 1,806 subjects who participated in the follow-up is shown in Table 1, with the age ranging from 18 years to 87 years, averaging 38.5 [30.0, 49.4] years; the proportion of female patients with Long COVID (50.87%) was higher than that of male patients (*p* = 0.023); among 1,806 patients, there were 1,319 (73.03%) patients who had received no more than two doses of COVID-19 vaccine in the past, showing a significant difference with those receiving more than 2 doses of COVID-19 vaccine (*p* = 0.001); statistical analysis of smoking history, allergy history, readmission, first symptoms, and total score of the questionnaire were statistically significant (*p* < 0.05), suggesting a correlation with Long COVID. There were no significant differences in age, past medical history and length of hospital stay (*p* > 0.05).

**Table 1:** Basic information of 1806 COVID-19 patients n(%)

Variables	Total(n = 1806)	Long Covid		*χ*^2^/Z	*P*
		Yes(n = 403)	No(n = 1403)		
Patients Sources				46.116	<0.001^*^
Changchun Infectious Disease Hospital	276	42(10.42)	234(16.68)		
Changchun Tongyuan Shelter Hospital	860	154(38.21)	706(50.32)		
Affiliated Hospital to Changchun University of Chinese Medicine	670	207(51.37)	463(33.00)		
Sex				5.152	0.023^*^
Women	829	205(50.87)	624(44.48)		
Men	977	198(49.13)	779(55.52)		
Age	38.5[30.0,49.4]	39.0[30.0,49.0]	38.3[30.0,49.7]	-0.018	0.986
Smoking				19.251	<0.001^*^
Smoking	632	104(25.81)	528(37.63)		
No smoking	1174	299(74.19)	875(62.37)		
Past history				0.141	0.707
Yes	366	79(19.60)	287(20.46)		
No	1440	324(80.40)	1116(79.54)		
Allergy history				4.504	0.034^*^
Yes	135	40(9.93)	95(6.77)		
No	1671	363(90.07)	1308(93.23)		
Readmission status				4.146	0.042^*^
Readmission	29	11(2.73)	18(1.28)		
No readmission	1777	392(97.27)	1385(98.72)		
Length of hospitalization				1.491	0.222
≤ 14 days	1259	315(78.17)	1060(75.55)		
> 14 days	414	63(15.63)	256(18.25)		
COVID-19 vaccine inoculation times				12.113	0.001^*^
≤ 2 doses	1319	267(66.25)	1052(74.98)		
> 2 doses	487	136(33.75)	351(25.02)		
First symptoms				9.108	0.003^*^
Yes	713	133(33.00)	580(41.34)		
No	1093	270(67.00)	823(58.66)		
Total questionnaire score	0[0,0]	0[0,2]	0[0,0]	-14.984	<0.001*

Note: *p < 0.05, the difference is statistically significant

### Analysis of long COVID influencing factors

Of the 1,806 patients from the three COVID-19 designated hospital, 403 subjects reported they had Long COVID, and the results of binary logistic regression analysis of influencing factors of Long COVID showed that, smoking history (OR (95%CI) = 0.551 (0.425–0.714), *p* < 0.001, taking never smoking as a reference), allergy history (OR (95%CI) = 1.618 (1.086–2.413), *p*-value 0.018, taking no allergy as a reference), first symptoms (OR(95%CI) = 0.636 (0.501–0.807), *p* < 0.001, taking no first symptoms as reference) and COVID-19 vaccine inoculation times (OR (95%CI) = 1.517 (1.190–1.933), *p*-value 0.001, taking COVID-19 vaccine inoculation times ≤ two doses as reference) could be considered as its influencing factors, as shown in Table 2.

**Table 2:** Binary logistic regression analysis of Long Covid influencing factors

Influencing factors	OR(95%CI)	*P*
Age (years)	0.996(0.988–1.004)	0.326
Sex		0.261
Men	1(Ref)	
Women	1.142(0.906–1.439)	0.261
Smoking history		<0.001^*^
No	1(Ref)	
Yes	0.551(0.425–0.714)	<0.001^*^
Allergy history		0.018^*^
No	1(Ref)	
Yes	1.618(1.086–2.413)	0.018^*^
First symptoms		<0.001^*^
No	1(Ref)	
Yes	0.636(0.501–0.807)	<0.001^*^
COVID-19 vaccine inoculation times		0.001^*^
≤ 2 doses	1(Ref)	
> 2 doses	1.517(1.190–1.933)	0.001^*^

Note: *p < 0.05, the difference is statistically significant

### First symptoms of patients on admission

Of the 1,806 patients, 713 (39.48%) had first symptoms on admission, of which the most common first symptoms included fever (512, 71.81%), cough (279, 39.13%) and dry or itchy throat (211, 29.59%), with the distribution these symptoms shown in Table 3.

**Table 3:** Table of frequency of first symptoms in 713 COVID-19 patients (n)

First symptoms	Total(n)
Fever	512
Cough	279
Dry throat/itchy throat	211
Dry mouth	37
Body pain	36
Coughing up phlegm	33
Headache	24
Nasal congestion	21
weakness	18
runny nose	12
spontaneous sweating	9
Cold	8
Sneezing	6
Shortness of breath/difficulty breathing	6
Diarrhea	5
Dizziness	1

### Long COVID symptoms

The statistical analysis of the frequency of symptoms in the 403 Long COVID patients revealed that the most common symptoms were persistent fatigue (68 cases), amnesia (61 cases), insomnia (50 cases) and excessive sweating (50 cases), with the distribution of these symptoms shown in Table 4.

**Table 4:** Table of frequency of Long Covid symptoms in 403 long Covid patients (n)

Long Covid symptoms	Total(n)		Long Covid symptoms	Total(n)
Constant fatigue	68		Constipation	9
amnesia	61		Fever	9
Insomnia	50		Weight loss	9
excessive sweating	50		Hearing problems	8
Depressed mood	45		Nausea	8
Anxiety	44		Diarrhea	8
Chest tightness	42		Bitterness in the mouth	7
Weakness in the limbs	40		Persistent muscle pain	7
Dizziness	32		Discomfort after exercise	7
Poor concentration	29		Sneezing	6
Dry/sore throat	28		Vomiting	6
Loss of sense of smell	27		Stomach pain	6
Decreased sense of taste	27		Bloating	6
Fear of cold	27		Urination problems	6
Pain/swelling in joints	21		Numbness or tingling	5
Runny nose	20		Dysmenorrhea	5
Sputum production	20		Sexual dysfunction	5
Itchy throat	20		Ringing in the ears	4
Persistent dry cough	19		Breathing pains	4
Heart palpitations	19		Abnormal walking posture/easy to fall	3
Shortness of breath	18		Mucous in the mouth	3
Loss of interest/joy	17		abdominal pain	3
Fear of heat	17		Slow movement	3
Chest pain	16		Behavioral changes	2
Nasal congestion	16		Twitching of extremities	2
Loss of appetite	16		Muscle stiffness	2
Vision problems	14		Edema	2
Heavy head and body	14		Hallucinations (seeing or hearing things that others cannot see or hear)	1
Persistent headache	13		Fainting/interruptions	1
Dry mouth/thirst	13		Seizures	1
Skin rash	13		Swallowing problems	1
Body aches and pains	12		Swelling of the ankles	1
Drowsiness	10		Localized skin lesions	1

## Discussion

It has been three years since the outbreak of COVID-19.Omicron and its subvariants are now the main viral variants, of which the pathogenicity has decreased, but the danger and impact of COVID-19 should never be ignored or underestimated [12]. COVID-19 patients usually recover with a range of symptoms in various systems of varying duration, leading to the development of Long COVID. It is estimated that approximately 65 million people worldwide suffer from Long COVID [13]. Long COVID will have an impact on the work and life of patients. Studies have shown that although COVID-19 patients can return to work after recovery, their health status is poorer than that of uninfected individuals [14]. It was found in a study that 45.2% of COVID-19 patients had their work hours reduced during the follow-up period and 22.3% did not work during the survey period due to their illness [7]. A study also showed that COVID-19 patients become less well after recovery and are prone to suffer from Long COVID, with some impact on exercise, work and life [8]. COVID-19 patients develop various sequelae after recovery and rarely recover completely from acute infections. Some sequelae may last for several years, or even exist for a lifetime, greatly increasing the medical and economic burden on patients and posing a serious threat to their physical health [15, 16]. Therefore, the study on the influencing factors associated with Long COVID under the influence of the Omicron variant and the development of better health management strategies for patients have become a hot and difficult problem.

Long COVID affects a wide range of populations all over the world. A study in the UK has shown that more than 50% of patients will be affected by Long COVID six months after discharge from hospital [17]. National studies have shown that patients may have one or more prolonged symptoms during follow-up, which may fluctuate and recur [14, 18, 19]. In addition, some studies have founded Long COVID in mildly ill and asymptomatic patients [20–22]. In conclusion, COVID-19 patients are at risk of Long COVID after recovery, which is manifested as one or more similar prolonged symptoms and has a serious impact on their daily life [23–25]. Therefore, the analysis of their clinical characteristics and the influencing factors can lead to better health management strategies for patients.

In this study, we analyzed the effects of patients’ basic information and clinical symptoms on Long COVID, and the results showed that among the 1,806 COVID-19 patients included, 403 were Long COVID patients, accounting for 22.31%, which was similar to previous research results. The results of the meta-analysis showed that up to 20% of outpatients would have Long COVID, and the morbidity of Long COVID was 54% in inpatients and approximately 34% in non-inpatients [26, 27]. Among the three COVID-19 designated hospitals included in this study, there was a certain correlation between the source of patients and the development of Long COVID, which might be associated with the different degrees of disease of patients admitted to the three hospitals at that time. The patients admitted to the designated hospital of the Affiliated Hospital to Changchun University of Chinese Medicine which is a Red Cross Hospital, so were generally critical patients, while the patients admitted to other two shelter hospitals were relatively mild cases. Among the Long COVID patients, there were 205 female patients, accounting for 50.87%, higher than the male patients (*p* = 0.023), which was similar to the results of previous studies, suggesting that female patients were at a higher risk of Long COVID and gender might be an independent risk factor for Long COVID [28–31]. In this study, we found that the first symptoms of patients on admission mainly included fever (921 cases, 50.10%), cough (652 cases, 36.10%) and dry or itchy throat (293 cases, 16.22%), and the most common Long COVID symptoms were persistent fatigue (68 cases), amnesia (61 cases), insomnia (50 cases), and excessive sweating (50 cases), which was similar to the results of many studies. It was founded [28] that among the 1,247 patients who participated in reporting symptoms, the most common symptom was fatigue (21.2%), followed by dyspnea (14.5%) and amnesia (9.1%). An analysis involving 199 individuals [32] showed that exertional dyspnea (47%) and fatigue (32%) were the most common symptoms during a 6-month follow-up survey. In addition, it was also reported [33] that among 384 patients, 69% had fatigue, 53% had persistent dyspnea, 34% had cough, and 14.6% had depression. Long COVID may be manifested as various symptoms, involving multiple systems, such as respiratory system, cardiovascular system, nervous system, and musculoskeletal system. The condition is complex and variable, exacerbating the patient’s psychological distress and medical burden, and affecting their daily life and work.

The survey and follow-up questionnaire of patients was formulated by the Global COVID-19 Clinical Platform Case Report Form for Post COVID condition to assess patients’ health status, including the following questions: Standing for long periods such as 30 min? Taking care of your household responsibilities? Learning a new task, e.g., learning how to get to a new place? Joining in community activities (e.g., festivities, religious, other)? Being emotionally affected by your health problems? Concentrating on doing something for ten minutes? Walking a long distance such as a kilometer (or equivalent)? Washing your whole body? Getting dressed? Dealing with people you do not know? Maintaining a friendship? Your day-to-day work/school? There were 12 items, and 5 options for each item, with scores of 0, 1, 2, 3, and 4, where 0 indicated no difficulty, 1 indicated mild difficulty, 2 indicated moderate difficulty, 3 indicated severe difficulty, and 4 indicated extreme difficulty or cannot do. The overall scores of patients were counted and recorded, and the results showed that there was a relationship between the overall questionnaire scores and Long COVID (*p* < 0.001), suggesting that Long COVID affected patients’ physical health status and daily life to different degrees. Multiple previous studies also pointed out that Long COVID affected patients’ work, life, and exercise [7, 8, 14–16], which were similar to our findings.

According to the binary logistic regression analysis of influencing factors for Long COVID, smoking history, first symptoms, allergy history and COVID-19 vaccine inoculation doses constituted the influencing factors for Long COVID, of which smoking history and first symptoms showed a negative association with Long COVID, while allergy history and COVID-19 vaccine inoculation doses showed a positive association with Long COVID. Smoking is a serious health hazard and one of the risk factors for many diseases. The World Health Organization has demonstrated that smokers are more likely to develop severe COVID-19, which can even lead to death in severe cases [34, 35]. However, some studies have also founded that smoking can reduce the risk of acquiring COVID-19. A retrospective cohort study [36] in 2.4 million subjects showed that people who smoked had a lower risk of acquiring COVID-19 compared to nonsmokers, and their risk of developing severe COVID-19 was also reduced. A meta-analysis [37] showed that smoking was a protective factor for positive detection of COVID-19, which can reduce the risk of positive detection (risk ratio [RR] = 0.74, 95% CI: 0.58–0.93). A study [38] of seafarers exposed to novel coronavirus also showed that those who smoked heavily were less likely to be infected. A large study in the UK noted that smoking was associated with a lower mortality rate of COVID-19 (OR = 0.88) [39]. The reason why smoking can reduce the risk of COVID-19 may be related to nicotine, the main substance in tobacco. Studies have pointed out that nicotine has certain anti-inflammatory effects and also binds to angiotensin converting enzyme 2 cell membrane proteins, downregulating the expression of nasal angiotensin converting enzyme 2 and other receptors for novel coronavirus infection, which in turn reduces the risk of COVID-19 and provides a certain protective effect [40–43]. The results of this study suggest there is a negative association between smoking and Long COVID, but the relationship between smoking and Long COVID is still somewhat controversial and needs to be verified by further studies with large samples.

The results of this study suggest that there is a negative association between first symptoms and Long COVID, which may be associated with the different severity of the disease in the patients included in the study and the pathogenic characteristics of the Omicron variant. Among the 1,806 patients included in this study, 713 patients had first symptoms on admission, of which only 133 had Long COVID, accounting for 18.65%, and the Omicron variant is weakly pathogenic and mostly mild or asymptomatic, which may explain the negative association between first symptoms and Long COVID, as previous studies have also pointed out that asymptomatic COVID-19 patients are likely to have Long COVID. Studies have shown that many Long Covid sequelae may occur in mild or even asymptomatic patients [44]. The study of Ma et al. also showed that asymptomatic COVID-19 patients might have symptoms of Long COVID, such as fatigue, cough, and loss of taste or smell [45].

Vaccination is an important preventive strategy to protect against diseases, and timely inoculation of COVID-19 vaccine can reduce the morbidity of COVID-19 and protect people’s health to some extent [46–49]. The results of this study showed that there was a positive association between Long COVID and COVID-19 vaccine inoculation doses, namely, the risk of Long COVID in patients who received > two doses of COVID-19 vaccine were lower compared with that in those who received ≤ two doses of COVID-19 vaccine. Recent studies have also shown that Long COVID may also occur in patients who received COVID-19 vaccine, and some studies have shown that approximately 2-10% of vaccinated COVID-19 patients have symptoms corresponding to Long COVID [26] and that 4.2-5.0% of patients with three doses of vaccination developed symptoms of Long COVID 12 weeks after infection [50]. According to the findings of numerous studies, although various brands of COVID-19 vaccines can still provide varying degrees of immune protection against Omicron variant, the immune escape capacity of various sublines of Omicron variant is also enhanced to varying degrees, and the protective effect of vaccination is significantly diminished, thereby making many patients still infected with Omicron variant after two and three doses of COVID-19 vaccine [51–55].

The results of this study showed there was a positive association between allergy history and Long COVID. It has been shown [56, 57] that patients with allergic diseases such as allergic rhinitis, hay fever, and eczema have a substantially reduced risk of COVID-19, which may be due to the fact that allergic reactions can reduce angiotensin converting enzyme 2 expression, which in turn hinders SARS-COV-2 receptor gene expression in humans. However, a recent study showed that subjects with immunocompromised disease had more severe COVID-19, longer hospital stays and an increased risk of fatal outcome of COVID-19 [58]; and the risk of Long COVID was relatively elevated in patients with allergy history and depressed immune function, which would be further compromised after infection with COVID-19, making it difficult to fight the virus.

## Conclusions

In conclusion, in this study, we initially analyzed the clinical characteristics of patients infected with Omicron variant of COVID-19 in Changchun in March 2022. The first symptoms on admission mainly included fever, cough and dry or itchy throat, the most common symptoms of Long COVID were persistent fatigue, amnesia, insomnia and excessive sweating, and female patients were at higher risk of Long COVID. Smoking history, COVID-19 vaccine inoculation doses, first symptoms and allergy history constituted the influencing factors associated with Long COVID, of which smoking history and first symptoms showed a negative association with Long COVID, while allergy history and COVID-19 vaccine inoculation doses showed a positive association with Long COVID. However, this study has some limitations, which lied in that we only performed telephone follow-up for the included patients in this study, and laboratory indexes and imaging examinations were not acquired, and this study was also regional. Furthermore, there are some potential biases such as the patients’ past health status were not clear. And there are also insufficiencies in the data collection of patient behavior, healthcare pattern, and subjective symptoms. Therefore, more well designed studies are needed to enrich the results of this study in the future.

### Electronic supplementary material

Below is the link to the electronic supplementary material.

Supplementary Material 1

Supplementary Material 2

## Data Availability

The datasets used and/or analysed during the current study are available from the corresponding author on reasonable request.

## Abbreviations

COVID-19: Coronavirus disease 2019
Long-COVID: Long-term sequalae of COVID-19
SARS-CoV-2: Severe acute respiratory syndrome coronavirus 2
CI: Confidence interval
OR: Odds ratio
