# Supplementary material for: Analysis of influencing factors on long COVID in COVID-19 patients infected with omicron variant three months after discharge: a cross-sectional study
Source: BMC Infect Dis. 2024 Jan 2;24:36. doi: 10.1186/s12879-023-08947-w (PMC10763267; doi:10.1186/s12879-023-08947-w)
Supplement: Supplementary file 1 — Supplementary Material 1 [file 12879_2023_8947_MOESM1_ESM.docx]

Table S1 Characteristics of comorbidities in 1806 covid-19 patients (n)

| Variables | Total(n=1806) | Long Covid | |
| --- | --- | --- | --- |
|  |  | Yes(n=403) | No(n=1403) |
| Hypertension | 154 | 30 | 124 |
| Diabetes | 65 | 12 | 53 |
| Coronary atherosclerotic  heart disease | 52 | 14 | 38 |
| Adiposis hepatica | 40 | 9 | 31 |
| Thyroid disease | 33 | 9 | 24 |
| Gallbladder disease | 18 | 5 | 13 |
| Hyperlipoidemia | 18 | 4 | 14 |
| Cerebral ischemia | 17 | 2 | 15 |
| Chronic renal disease | 7 | 3 | 4 |
| Chronic liver disease | 7 | 3 | 4 |
| Immunodeficiency | 7 | 2 | 5 |
