# Supplementary material for: Analysis of influencing factors on long COVID in COVID-19 patients infected with omicron variant three months after discharge: a cross-sectional study
Source: BMC Infect Dis. 2024 Jan 2;24:36. doi: 10.1186/s12879-023-08947-w (PMC10763267; doi:10.1186/s12879-023-08947-w)
Supplement: Supplementary file 2 — Supplementary Material 2 [file 12879_2023_8947_MOESM2_ESM.pdf]

Global COVID-19 Clinical Platform
Case Report Form (CRF) for Post COVID condition (Post COVID-19 CRF)

The WHO has established a Global Clinical Data Platform[1] of COVID-19 and invites all Member States and health facilities to report anonymised patient-level clinical information to the WHO platform using standardized Case Report Form (CRF):

- *Core CRF* captures clinical information of individuals hospitalized for COVID-19
- *Core-P CRF* has information of pregnant women hospitalized for COVID-19
- *MIS-CRF* has information related to multisystemic inflammatory syndrome in children and adolescents temporally related to COVID-19
- *Post COVID-19 CRF*, designed to build upon the Core CRF and assess the medium- and long-term sequelae of COVID-19

The Post COVID-19 CRF includes 3 modules:
Module 1 includes background demographic and clinical information of the acute episode of COVID-19.
Module 2 includes questions to help identifying patients who require further clinical evaluation.
Module 3 includes medical assessment and results of examinations, tests, or diagnosis made during the follow up visit. Based on results, patients should be referred for clinical care, or rehabilitation as per national protocols.

The Post COVID-19 CRF is intended to serve as: (i) A clinical tool that can be used by Member States to document the mid- and long-term sequelae of COVID-19. Uniformity in the follow up of patients could ensure that mid- and long-term clinical and rehabilitation needs are identified, and patients are provided the care they need; **(ii)** WHO is not necessarily recommending the comprehensive testing described in the CRF for all individuals; clinician judgement is required to select the test needed for clinical care. This CRF is a tool for gathering standardized information regarding the post COVID-19 condition through the WHO Clinical Data Platform. Such data collation and its analysis would improve national and global knowledge of the consequences of COVID-19, inform further public health responses and prepare for large investigational studies.
Criteria for completion of Post COVID-19 CRF: Variables' dictionary available on WHO website[1] support data entry. The CRF can be administered either as part of routine follow up or at a specific time point to any patient in the post-acute phase of COVID-19, regardless of hospitalization. While it is suggested to prioritize the completion of the CRF for patients *who were hospitalized for a severe or critical* episode of COVID-19, the CRF should be administered, where possible, also to patients who suffered from COVID-19, including those with mild or moderate illness, and who *received care either at home or in a hospital setting.*
Time-points for administration: The form can be completed any time during follow up after an acute episode of COVID-19. However, to support standardization and data comparability, it should preferably be completed 4 to 8 weeks and 6 months after hospital discharge from the acute ward or after acute illness for individuals who have not been hospitalized. In case of persistent symptoms/signs after hospital discharge or after acute illness, it is recommended to complete the CRF at 3-month intervals, for as long as needed, or at 6 months interval, if no symptoms persist (see figure below).
Mode of administration:
Module 1-2: face-to-face administration and completion by a health care worker is preferred. However, when this is not possible, the form can be either self-administered, or completed remotely (online or through telephone) by the caregiver. For children, the form should be completed by the primary caregiver (preferred) or by the legal guardian.
Module 3: face-to-face administration and completion by a health care worker.
Module 1 needs to be completed only once during the first follow up visit, while Modules 2 and 3 should be completed at every follow up visit.
General guidance: Please contact **COVID_ClinPlatform@who.int** if you need assistance with data entry, if you have any query on the CRF, and to let us know that you are using the forms.

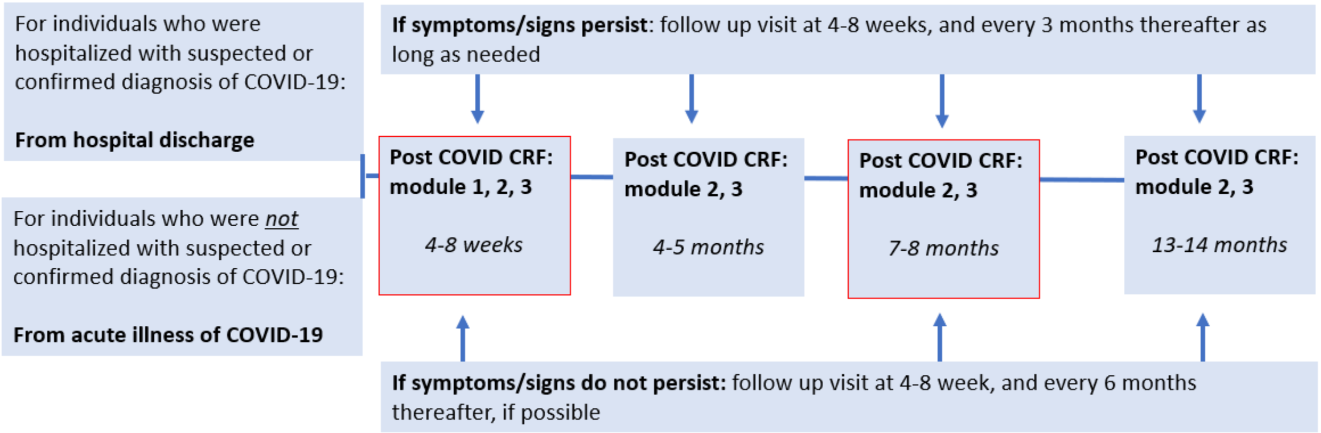

[1] https://www.who.int/teams/health-care-readiness-clinical-unit/covid-19/data-platform

World Health Organization

PARTICIPANT ID[2] I__I I__I I__I I__I I__I -- I___I I___I I___I I___I

Module 1: Background demographical and epidemiological information

This module is completed by ☐patient ☐caregiver (in case of children) ☐health care worker
Facility name of follow up visit (if applies) ______________________________ Country__________________
Date of module 1 completion: [_D_][_D_]/[_M_][_M_]/[_Y_][_Y_][_Y_][_Y_]

1.1 Acute episode of COVID-19 information (first episode, in case of re-infection)

Does the patient have a WHO Rapid Core CRF Participant ID?
If Yes, report PARTICIPANT ID of CORE CRF I___I I___I I___I I___I I___I -- I___I I___I I___I I___I

1.2 Demographics

Sex at Birth: ☐Male ☐Female ☐Not specified
Age: [ ][ ][ ] years; OR [ ][ ] months [ ][ ] days
Height (Length): [ ][ ][ ] cm
Weight: [ ][ ][ ] kg
Highest level of education completed? ☐No schooling or never completed any grade
☐Elementary school ☐Vocational school ☐Secondary school ☐University
In the last 3 years, has the participant ever stayed overnight in a hospital, rehabilitation facility, or long-term care facility? ☐Yes, a hospital ☐Yes, a rehabilitation facility ☐Yes, a long-term care facility ☐All ☐No ☐Unknown
Was the participant a long-term care facility resident prior to initial COVID-19 diagnosis?
☐Yes ☐No ☐Unknown
Ethnicity/background: ☐Asian ☐Black ☐White ☐Mixed ☐Arab ☐Latino ☐Other ☐Unknown
Smoking: ☐Current ☐Former ☐Never ☐Unknown
Substance abuse: ☐Yes ☐No ☐Unknown; If yes:☐Alcohol ☐Drug ☐Other
Was this participant employed as a health care worker or laboratory staff since Jan 1st, 2020?
☐Yes ☐No ☐Unknown

Pregnancy information

Was the participant **pregnant** during the **acute** illness of COVID-19? ☐Yes ☐No ☐Unknown; **If yes**, gestational weeks at COVID-19 diagnosis/clinical suspicion: [_][_] weeks ☐Unknown;
If **pregnant** during the **acute** illness, outcome of pregnancy? ☐Miscarriage ☐Induced abortion ☐Still birth ☐Live birth ☐Still pregnant;
If **pregnant** during the **acute** illness, and **currently not pregnant**: gestational age at the time of delivery/abortion? [ ][ ] weeks;
If delivered, mode of delivery? ☐Vaginal ☐Assisted vaginal ☐Caesarean section;
Is the participant currently pregnant? ☐Yes ☐No ☐Unknown; **If yes, gestational weeks** [ ][ ]Weeks ☐Unknown;
If recently pregnant, is the participant currently breastfeeding? ☐Yes ☐No ☐ Unknown

1.3 Pre-existing conditions in the year prior to your acute illness of COVID-19:

In the year prior to the acute illness of COVID-19, has the participant been diagnosed with any of the following conditions?

**Asplenia:**	☐Yes ☐No ☐Unknown;
**Cancer:**	☐Yes ☐No ☐Unknown;
**Chronic heart disease (not hypertension):**	☐Yes ☐No ☐Unknown;
**Chronic kidney disease**:	☐Yes ☐No ☐Unknown;
**Chronic liver disease:**	☐Yes ☐No ☐Unknown;
**Chronic lung disease:**	☐Yes ☐No ☐Unknown;
**Chronic neurological disorder:**	☐Yes ☐No ☐Unknown;
If Yes, specify:	☐Dementia ☐Stroke ☐Multiple Sclerosis ☐Parkinson's Disease;
**Diabetes:**	☐Yes ☐No ☐Unknown;
**HIV:**	☐Yes ☐No ☐Unknown; If Yes, was on ART? ☐Yes ☐No ☐Unknown;
If Yes, what regimen?	☐Protease inhibitor-based ART; ☐NNRTI-based ART ☐Integrase inhibitor-based ART; Last viral load test: ________________copies/ml; Last CD4 cell count: [ ][ ] [ ][ ] cells/mm^3;
**Hypertension:**	☐Yes ☐No ☐ Unknown;
If Yes, did the participant receive medication?	☐Yes ☐No ☐Unknown;
**Immunodeficiency:**	☐Yes ☐No ☐Unknown;
**Mental health conditions:**	☐Yes ☐No ☐Unknown.
If Yes, specify:	☐psychoses ☐depression ☐anxiety;
**Obesity (BMI>30):**	☐Yes ☐No ☐Unknown;
**Tuberculosis:**	☐Yes ☐No ☐Unknown; If yes ☐Active ☐Previous;
**Any other condition:**	☐Yes ☐No; **If yes, specify**______________

World Health Organization

1.4 Diagnosis of acute illness of COVID-19 (first episode, in case of re-infection)
Date of onset of symptoms of **acute** COVID-19: [_D_][_D_]/[_M_][_M_]/[_Y_][_Y_][_Y_][_Y_];<br>Did the participant receive a **diagnosis** of COVID-19 by a health care worker during the **acute illness**?<br>□Yes □No □Unknown;<br>Did the participant have a **diagnostic test?** □Yes □No □Unknown;<br>If yes, complete the 3 questions below:<br>Did the participant have a **PCR test** during the acute illness?<br>□Yes, positive □Yes, negative □Not performed □Unknown;<br>**If positive,** date of positive PCR test: [_D_][_D_]/[_M_][_M_]/[_Y_][_Y_][_Y_][_Y_]<br>Did the participant have an **antigen test** (rapid test) during acute illness?<br>□Yes, positive □Yes, negative □Not performed □Unknown;<br>**If positive,** date of positive antigen test: [_D_][_D_]/[_M_][_M_]/[_Y_][_Y_][_Y_][_Y_]<br>Did the participant have an **antibody test** during/after the acute illness?<br>□Yes, positive □Yes, negative □Not performed □Unknown;<br>**If positive,** date of positive antibody test: [_D_][_D_]/[_M_][_M_]/[_Y_][_Y_][_Y_][_Y_]<br>Please grade the **severity of acute illness** of COVID-19 based on WHO criteria described in the table below.<br>Please tick the classification that applies: □Mild □Moderate □Severe □Critical □Unknown

WHO Clinical Classification	Based on available clinical records	Based on self-report, if clinical records are not available
**Mild**	No hypoxia or pneumonia	Did not receive oxygen
**Moderate**	Clinical signs of non-severe pneumonia *AND* SpO2≥90% on room air	
**Severe**	**Adults/adolescents**: Clinical signs of severe pneumonia *AND* SpO2 <90% on room air; *OR* RR > 30 breaths/min<br>**Children**: Clinical signs of severe pneumonia *AND at least one of the following:* central cyanosis; *OR* SpO2 < 90%; *OR* severe respiratory distress (e.g. fast breathing, grunting, very severe chest indrawing); *OR* general danger sign(s) (inability to breastfeed or drink, lethargy or unconsciousness, convulsions)	Received oxygen (or told you they needed it, but it was not available)
**Critical**	ARDS; *OR* sepsis/septic shock; *OR* pulmonary embolism, acute coronary syndrome, acute stroke;<br>*OR* Multi-Inflammatory Syndrome in Children and adolescents temporally related to COVID-19	Received invasive ventilation (or max available respiratory support)

1.5 Clinical management while unwell during the acute COVID-19 episode
**Highest level of care received during the acute episode?** □Admitted to the hospital □Self-care/Over-the-counter □Treated at home/Telemedicine □Outpatient □Unknown;<br>**If admitted to the hospital:**<br>Date of hospital admission: [_D_][_D_]/[_M_][_M_]/[_Y_][_Y_][_Y_][_Y_];<br>Date of hospital discharge: [_D_][_D_]/[_M_][_M_]/[_Y_][_Y_][_Y_][_Y_];<br>Duration of hospital stay (total) during acute episode of COVID-19: I___I I___I I___I days;<br>Was the participant admitted to Intensive Care Unit or lower dependency unit? □Yes □No □Unknown;<br>**Did the participant receive oxygen therapy during the acute illness?** □Yes □No □Unknown<br>If yes, did the participant receive invasive ventilation (a machine that breaths for you)? □Yes □No □Unknown<br>If yes, did the participant receive non-invasive ventilation (e.g. mask providing pressurized air and oxygen to help you breathing)? □Yes □No □Unknown;
**Treatment:** Did the participant receive **treatment for COVID-19**? □Yes □No;
**If yes**, complete section below:<br>**Antibiotic received?** □Yes □No □Unknown;<br>If yes, specify**:** □ Macrolides (e.g. Azithromycin, clarithromycin) □ Fluoroquinolones (e.g. ciprofloxacin, levofloxacin □3rd and 4rd generation Cephalosporins (e.g. ceftriaxone, cefotaxime, ceftazidime, cefepime) □Carbapenems (e.g imipenem, meropenem) □Piperacillin + Tazobactam □Amoxicillin-clavulanate □ Cotrimoxazole □Other antibiotics ___________________________;<br>Duration of antibiotics therapy (days): [__][__]<br>**Antithrombotic/anticoagulation drugs received?** □Yes □No □Unknown;<br>If yes specify**:** □Unfractionated heparin □Low molecular weight heparin □Warfarin □ Direct oral anticoagulant □Other ___________________________; Dose: ☐Preventive dose ☐Therapeutic dose<br>**Antiviral drugs received?** □Yes □No □Unknown;<br>If yes, specify: □Lopinavir/Ritonavir □Darunavir +/- cobicistat □Remdesivir □Favipiravir □Acyclovir/Ganciclovir □Oseltamivir □Other ___________________________;

World Health Organization

1.5 Clinical management while unwell during the acute COVID-19 episode *continuation*	
**Blood-derived products received?** □Yes □No □Unknown;	
If yes, specify: □IV immune globulin □Convalescent plasma □Other____________________________;	
**Chloroquine/hydroxychloroquine received?** □Yes □No □Unknown;	
If Yes, purpose: □malaria prophylaxis □COVID-19 prophylaxis; □COVID-19 treatment	
**Experimental agents:**	
Ivermectin received?	□Yes □No □Unknown
Interferon received?	□Yes □No □Unknown
Eculizumab received?	□Yes □No □Unknown
Pytotherapy received?	□Yes □No □Unknown
IL-1 Antagonists received?	□Yes □No □Unknown;
If Yes, specify:	□Anakinra □Canakinumab; □Other IL-1 antagonist;
IL-6 Antagonists received?	□Yes □No □Unknown;
If Yes, specify:	□Siltuximab □Sarilumab □Tocilizumab □Other IL-6 antagonist;
Kinase Inhibitors received?	□Yes □No □Unknown;
If Yes, specify:	□Acalabrutinib □Ibrutinib □Zanubrutinib □Baricitinib □Ruxolitinib □Tofacitinib □Ruxolitinib; □Other Kinase inhibitors;
Neutralizing monoclonal antibodies received? □Yes □No □Unknown; If Yes, specify: _____________________;	
Other agents:	□Yes □No □Unknown; If Yes, specify: ___________________________________;
**Steroids received?** □Yes □No □Unknown;	
If yes specify: □Dexamethasone □Hydrocortisone □Prednisone □Methylprednisolone □Other _____________	
Duration of steroid therapy (days): [__][__] Dose: _____________ Route: □Oral □Intravenous □Inhaled	

World Health Organization

PARTICIPANT ID[2] I___I I___I I___I I___I I___I – I___I I___I I___I I___I

Module 2. Follow up interview

This module is completed by ☐patient ☐caregiver (in case of children) ☐health care worker
Date of follow up interview: [_D_][_D_]/[_M_][_M_]/[_Y_][_Y_][_Y_][_Y_]
Country ________________ City: __________ Facility name (if applies) _______________________________

**2.1 Hospital admission after the acute illness of COVID-19**
Was the participant **admitted to the hospital** for a possible **complication** of COVID-19 **after the acute illness?** ☐Yes ☐No ☐Unknown; If yes, date of (re)admission [_D_][_D_]/[_M_][_M_]/[_Y_][_Y_][_Y_][_Y_] and please specify type of complication in section 3.5

**2.2 Reinfection**
Did the participant experience a second episode/reinfection with SARS-CoV-2? ☐Yes ☐No ☐Unknown<br>If yes, date of **second positive PCR:** [_D_][_D_]/[_M_][_M_]/[_Y_][_Y_][_Y_][_Y_]<br>What is the highest level of care received during the second episode? ☐Admitted to the hospital ☐Self-care/Over-the-counter ☐Outpatient/Telemedicine ☐Community facility ☐Unknown

**2.3 Vaccination status for Covid-19**
Did the patient receive a Covid-19 vaccine? ☐Yes ☐No ☐Unknown<br>If yes, number of doses received: ☐1 ☐2 ☐Unknown<br>Product name of COVID-19 vaccine dose 1:<br>☐Moderna ☐Pfizer-BioNTech ☐AstraZeneca ☐Janssen ☐Novavax ☐Other ☐Unknown;<br>Date of vaccine dose 1: [D][D]/[M][M]/[Y][Y][Y][Y]<br>Product name of COVID-19 vaccine dose 2:<br>☐Moderna ☐Pfizer-BioNTech ☐AstraZeneca ☐Janssen ☐Novavax ☐Other ☐Unknown;<br>Date of vaccine dose 2: [D][D]/[M][M]/[Y][Y][Y][Y]<br>Source of information: ☐Documented Evidence (Vaccine card/Vaccine Passport/Facility based record/other); ☐Recall

**2.4 Occupational status**
Is there a change in the duration (hours) of working or schooling as compared to before acute illness of COVID-19? ☐Yes ☐No ☐Unknown;<br>**If yes, specify:** ☐Working/schooling time increased ☐Working/schooling time decreased ☐Stopped working or schooling since COVID-19 ☐Unknown;<br>**If less or not working or schooling**, what is the reason? ☐Poor health ☐New caring responsibility ☐Work or school less or not available due to COVID-19 restrictions ☐Other ☐Prefer not to say ☐Unknown

2.5 Functioning (*do not need complete this section for children <15yrs*)

Ability to self-care: ☐Same as before COVID-19 ☐Worse ☐Better ☐Unknown

**Think back over the past 7 days.**<br>**How much difficulty has the participant had with the following:**	**Score:**<br>**0** No Difficulty<br>**1** Mild Difficulty<br>**2** Moderate Difficulty<br>**3** Severe Difficulty<br>**4** Extreme Difficulty or Cannot do	**Compared to before COVID-19, are you better/worse/same?**		
		Better	Worse	Same
Standing for long periods such as 30 minutes?				
Taking care of your household responsibilities?				
Learning a new task, e.g. learning how to get to a new place?				
Joining in community activities (e.g. festivities, religious, other)?				
Being emotionally affected by your health problems?				
Concentrating on doing something for ten minutes?				
Walking a long distance such as a kilometre (or equivalent)?				
Washing your whole body?				
Getting dressed?				
Dealing with people you do not know?				
Maintaining a friendship?				
Your day-to-day work/school?				
**TOTAL score**				

If other scales were used: Name of the scale: ________________________________Score_[_ _][_ _]/[_ _][_ _]

[2] **Participant ID**: obtain the 4-digit **site code** by contacting COVID_ClinPlatform@who.int. Enter a 5-digit **patient number** (e.g. 00001, 00002, etc) and record the information in a logbook

World Health Organization

2.6 Incidence of symptoms after acute illness of COVID-19

Did the participant experience any of the following symptoms after the acute illness of COVID-19/ since hospital discharge for COVID-19, that were **not experienced** before the acute episode of COVID-19? ☐Yes ☐No ☐Unknown;
If yes, please respond to questions below:
Anxiety: ☐Yes, but not present anymore ☐Yes, still present ☐Yes, intermittent ☐No ☐Unknown;
Behaviour change: ☐Yes, but not present anymore ☐Yes, still present ☐Yes, intermittent ☐No ☐Unknown;
Can't move and/or feel one side of body or face: ☐Yes, but not present anymore ☐Yes, still present ☐Yes, intermittent ☐No ☐Unknown;
Chest pain: ☐Yes, but not present anymore ☐Yes, still present ☐Yes, intermittent ☐No ☐Unknown;
Constipation: ☐Yes, but not present anymore ☐Yes, still present ☐Yes, intermittent ☐No ☐Unknown;
Depressed mood: ☐Yes, but not present anymore ☐Yes, still present ☐Yes, intermittent ☐No ☐Unknown;
Diarrhoea: ☐Yes, but not present anymore ☐Yes, still present ☐Yes, intermittent ☐No ☐Unknown;
Dysmenorrhea ☐Yes, but not present anymore ☐Yes, still present ☐Yes, intermittent ☐No ☐Unknown;
Dizziness/light headedness: ☐Yes, but not present anymore ☐Yes, still present ☐Yes, intermittent ☐No ☐Unknown;
Fainting/blackouts: ☐Yes, but not present anymore ☐Yes, still present ☐Yes, intermittent ☐No ☐Unknown;
Fever: ☐Yes, but not present anymore ☐Yes, still present ☐Yes, intermittent ☐No ☐Unknown;
Forgetfulness: ☐Yes, but not present anymore ☐Yes, still present ☐Yes, intermittent ☐No ☐Unknown;
Jerking of limbs: ☐Yes, but not present anymore ☐Yes, still present ☐Yes, intermittent ☐No ☐Unknown;
Joint pain/swelling: ☐Yes, but not present anymore ☐Yes, still present ☐Yes, intermittent ☐No ☐Unknown;
Loss of appetite: ☐Yes, but not present anymore ☐Yes, still present ☐Yes, intermittent ☐No ☐Unknown;
Loss of interest/pleasure: ☐Yes, but not present anymore ☐Yes, still present ☐Yes, intermittent ☐No ☐Unknown;
Lumpy lesions: (purple/pink/bluish) **on toes/COVID toes:** ☐Yes, but not present anymore ☐Yes, still present ☐Yes, intermittent ☐No ☐Unknown;
Nausea/vomiting: ☐Yes, but not present anymore ☐Yes, still present ☐Yes, intermittent ☐No ☐Unknown;
Numbness or tingling: ☐Yes, but not present anymore ☐Yes, still present ☐Yes, intermittent ☐No ☐Unknown;
Pain on breathing: ☐Yes, but not present anymore ☐Yes, still present ☐Yes, intermittent ☐No ☐Unknown;
Palpitations: ☐Yes, but not present anymore ☐Yes, still present ☐Yes, intermittent ☐No ☐Unknown;
Persistent dry cough: ☐Yes, but not present anymore ☐Yes, still present ☐Yes, intermittent ☐No ☐Unknown;
Persistent fatigue: ☐Yes, but not present anymore ☐Yes, still present ☐Yes, intermittent ☐No ☐Unknown;
Problems hearing: ☐Yes, but not present anymore ☐Yes, still present ☐Yes, intermittent ☐No ☐Unknown;
Persistent headache: ☐Yes, but not present anymore ☐Yes, still present ☐Yes, intermittent ☐No ☐Unknown;
Persistent muscle pain: ☐Yes, but not present anymore ☐Yes, still present ☐Yes, intermittent ☐No ☐Unknown;
Post-exertional malaise: ☐Yes, but not present anymore ☐Yes, still present ☐Yes, intermittent ☐No ☐Unknown;
Problems passing urine: ☐Yes, but not present anymore ☐Yes, still present ☐Yes, intermittent ☐No ☐Unknown;
Problems seeing: ☐Yes, but not present anymore ☐Yes, still present ☐Yes, intermittent ☐No ☐Unknown;
Problem swallowing: ☐Yes, but not present anymore ☐Yes, still present ☐Yes, intermittent ☐No ☐Unknown;
Problems with balance: ☐Yes, but not present anymore ☐Yes, still present ☐Yes, intermittent ☐No ☐Unknown;
Problems with gait/falls: ☐Yes, but not present anymore ☐Yes, still present ☐Yes, intermittent ☐No ☐Unknown;
Reduced smell: ☐Yes, but not present anymore ☐Yes, still present ☐Yes, intermittent ☐No ☐Unknown;
Reduced taste: ☐Yes, but not present anymore ☐Yes, still present ☐Yes, intermittent ☐No ☐Unknown;
Ringing in ears: ☐Yes, but not present anymore ☐Yes, still present ☐Yes, intermittent ☐No ☐Unknown;
Seizures: ☐Yes, but not present anymore ☐Yes, still present ☐Yes, intermittent ☐No ☐Unknown;
Shortness of breath: ☐Yes, but not present anymore ☐Yes, still present;
If yes: ☐Present ☐ At rest ☐With activity; ☐Yes, intermittent ☐No ☐Unknown;
Skin rash: ☐Yes, but not present anymore ☐Yes, still present ☐Yes, intermittent ☐No ☐Unknown;
If yes, please tick all areas of the body that apply: ☐Face ☐Trunk (stomach or back) ☐Arms ☐Legs ☐Buttocks ☐Toes ☐Fingers;
Slowness of movement: ☐Yes, but not present anymore ☐Yes, still present ☐Yes, intermittent ☐No ☐Unknown;
Sleeping less: ☐Yes, but not present anymore ☐Yes, still present ☐Yes, intermittent ☐No ☐Unknown;
Sleeping more: ☐Yes, but not present anymore ☐Yes, still present ☐Yes, intermittent ☐No ☐Unknown;
Stiffness of muscles: ☐Yes, but not present anymore ☐Yes, still present ☐Yes, intermittent ☐No ☐Unknown;
Stomach pain: ☐Yes, but not present anymore ☐Yes still present ☐Yes, intermittent ☐No ☐Unknown;
Swollen ankles: ☐Yes, but not present anymore ☐Yes, still present ☐Yes, intermittent ☐No ☐Unknown;
Tremors: ☐Yes, but not present anymore ☐Yes, still present ☐Yes, intermittent ☐No ☐Unknown;
Trouble in concentrating: ☐Yes, but not present anymore ☐Yes, still present ☐Yes, intermittent ☐No ☐Unknown;
Weakness in limbs: ☐Yes, but not present anymore ☐Yes, still present ☐Yes, intermittent ☐No ☐Unknown;
Weight loss: ☐Yes, but not present anymore ☐Yes, still present ☐Yes, intermittent ☐No ☐Unknown;
The following questions should not be completed for children <15yrs:
Erectile dysfunction: ☐Yes, but not present anymore ☐Yes, still present ☐Yes, intermittent ☐No ☐Unknown;
Hallucinations (seeing or hearing things others don't see or hear): ☐Yes, but not present anymore ☐Yes, still present ☐Yes, intermittent ☐No ☐Unknown

PARTICIPANT ID[3] I___I I___I I___I I___I I___I – I___I I___I I___I I___I

Module 3: Clinical examinations, laboratory tests and diagnosis during follow up visit
This module should be completed by a health worker to report on examinations/tests undertaken during the current follow up visit. **Date of follow up visit:** [_D_][_D_]/[_M_][_M_]/[_Y_][_Y_][_Y_][_Y_]
Country ________________ City: __________ Facility name (if applies) ______________________

**3.1 Neurological examination**
Was a neurological examination performed? □Yes □No □Unknown;<br>**If yes**, findings were: □Normal □Abnormal □Unknown;<br>*If abnormal, select below the abnormalities that apply:*<br>**Aphasia**: □Yes □No □Unknown;<br>**Ataxia**: □Yes □No □Unknown;<br>**Confusion**, **disorientation or otherwise abnormal mental status**: □Yes □No □Unknown;<br>**Dysarthria**: □Yes □No □Unknown;<br>**Dystonia**: □Yes □No □Unknown;<br>**Facial weakness:** □Yes □No □Unknown;<br>**Hearing loss**: □Yes □No □Unknown;<br>**Hemiparesis**: □Yes □No □Unknown;<br>**Neuralgia:** □Yes □No □Unknown;<br>**Paraparesis**: □Yes □No □Unknown;<br>**Sensory Loss**: □Yes □No □Unknown;<br>**Tremor or abnormal movements**: □Yes □No □Unknown;<br>**Vision loss** (including ocular, field cut): □Yes □No □Unknown

**3.2 Radiographic examinations**
Did the participant perform any radiographic examination? □Yes □No □Unknown;<br>**If yes**, please specify type of exam and results:<br>**CT Scan Brain:** □Done □Not done □Unknown;<br>**If done:** □Normal □Abnormal, likely unrelated to COVID-19 □ Abnormal, likely related to COVID-19 □Abnormal, but unknown if related to COVID-19;<br>**CT Scan Chest:** □Done □Not done □Unknown;<br>**If done:** □Normal □Abnormal, likely unrelated to COVID-19 □Abnormal, likely related to COVID-19 □Abnormal, but unknown if related to COVID-19;<br>**Echocardiogram:** □Done □Not done □Unknown;<br>**If done:** □Normal □Abnormal, likely unrelated to COVID-19 □Abnormal, likely related to COVID-19 □Abnormal, but unknown if related to COVID-19;<br>**Lung ultrasound:** □Done □Not done □Unknown;<br>**If done:** □Normal □Abnormal, likely unrelated to COVID-19 □Abnormal, likely related to COVID-19 □Abnormal, but unknown if related to COVID-19;<br>**MRI Brain:** □Done □Not done □Unknown;<br>**If done:** □Normal □Abnormal, likely unrelated to COVID-19 □Abnormal, likely related to COVID-19 □Abnormal, but unknown if related to COVID-19;<br>**MRI Spine**: □Done □Not done □Unknown;<br>**If done:** □Normal □Abnormal, likely unrelated to COVID-19 □Abnormal, likely related to COVID-19 □Abnormal, but unknown if related to COVID-19;<br>**X-ray Chest:** □Done □Not done □Unknown;<br>**If done:** □Normal □Abnormal, likely unrelated to COVID-19 □Abnormal, likely related to COVID-19 □Abnormal, but unknown if related to COVID-19

[3] **Participant ID**: obtain the 4-digit **site code** by contacting COVID_ClinPlatform@who.int. Enter a 5-digit **patient number** (e.g. 00001, 00002, etc) and record the information in a logbook

World Health Organization

3.3 Blood tests			
Was a blood test done? Yes No Unknown;<br>**If yes**, specify type of test, date, and results from list below:			
**Albumin:**	Done Not done	Value:	g/L g/dL
**ALT/SGPT:**	Done Not done	Value:	IU/L
**Antithyroglobulin:**	Done Not done	Value:	IU/ml
**AST/SGOT:**	Done Not done	Value:	IU/L
**Creatine Kinase** MM:	Done Not done	Value:	IU/L UKAT/L
**Creatinine:**	Done Not done	Value:	mg/dL μmol/L
**C-reactive protein (CRP):**	Done Not done	Value:	mg/L
**D-Dimer:**	Done Not done	Value:	ng/mL μg/L
**Fasting Blood Glucose:**	Done Not done	Value:	mg/dL
**Ferritin:**	Done Not done	Value:	ng/mL μg/L
**Fibrinogen:**	Done Not done	Value:	g/L mg/dL
**Globular Filtration Rate:**	Done Not done	Value:	ml/min
**LDH:**	Done Not done	Value:	IU/L
**Lymphocytes:**	Done Not done	Value:	cells/μL cells/mm^3
**Thyroid peroxidase antibodies:**	Done Not done	Value:	U/ml
**Troponin:**	Done Not done	Value:	ng/mL μg/L
**TSH:**	Done Not done	Value:	mU/L
**Urea (BUN):**	Done Not done	Value:	g/L mg/dL mmol/L
**Coronavirus antibodies IgA:**	Done Not done	Value:	Pos Neg
**Coronavirus antibodies IgG:**	Done Not done	Value:	Pos Neg
**Coronavirus antibodies IgM:**	Done Not done	Value:	Pos Neg

3.4 Clinical Tests and Scales

Was a neurological test done? Yes No Unknown;
If yes, specify type of test and results from list below:
Addenbrooke's Cognitive Examination-III (ACE-III): Done Not done Unknown;
If done, score 0-100 [ ][ ][ ];
Cerebral Spinal Fluid examination: Done Not done Unknown;
If done: Normal Abnormal, likely unrelated to COVID-19 Abnormal, likely related to COVID-19 Abnormal, unknown if related to COVID-19 Unknown;
Electroencephalogram: Done Not done Unknown;
If done: Normal Abnormal, likely unrelated to COVID-19 Abnormal, likely related to COVID-19 Abnormal, unknown if related to COVID-19 Unknown;
Electromyogram: Done Not done Unknown;
If done: Normal Abnormal, likely unrelated to COVID-19 Abnormal, likely related to COVID-19 Abnormal, unknown if related to COVID-19 Unknown;

Hearing test: Done Not done Unknown;
If done: Normal Abnormal, likely unrelated to COVID-19 Abnormal, likely related to COVID-19 Abnormal, unknown if related to COVID-19 Unknown;

Mini-Mental State Examination (MMSE): Done Not done Unknown;
If done: score 0-30 [ ][ ];

Montreal Cognitive Assessment (MoCA): Done Not done Unknown;
If done: score 0-30 [ ][ ];

Nerve Conduction Studies: Done Not done Unknown;
If done: Normal Abnormal, likely unrelated to COVID-19 Abnormal, likely related to COVID-19 Abnormal, unknown if related to COVID-19 Unknown;

Vision test: Done Not done Unknown;
If done: Normal Abnormal, likely unrelated to COVID-19 Abnormal, likely related to COVID-19 Abnormal, unknown if related to COVID-19 Unknown;

Other tests performed: Done Not done Unknown;
If done: Name of the test ___________________ **Results:** Normal Abnormal Unknown

World Health Organization

3.4 Clinical Tests and Scales *continuation*

Was a cardiovascular test done? ☐Yes ☐No ☐Unknown;
If yes, specify type of test and results from list below:

Electrocardiogram: ☐Done ☐Not done ☐Unknown;
If done: ☐Normal ☐Abnormal;

6-Minute Walking Distance: ☐Done ☐Not done;
If done: [ ][ ][ ] metres;

Pulse rate at rest: [ ][ ][ ] beats/minute ☐ Unknown;

Other tests performed: ☐Done ☐Not done ☐Unknown;
If done: Name of the test ___________________ **Results:** ☐Normal ☐Abnormal ☐Unknown

Was a pulmonary test done? ☐Yes ☐No ☐Unknown;
If yes, specify type of test and results from list below:

Diffusing Capacity for Carbon Monoxide (DCLO) test: ☐Done ☐Not done; If done, [ ][ ][ ] %;

Is the patient receiving supplemental oxygen? ☐Yes ☐No ☐Unknown;

MRC dyspnoea scale: ☐Score 1 ☐Score 2 ☐Score 3 ☐Score 4 ☐Score 5 ☐Unknown;

Pulmonary Function Test: ☐Done ☐Not done ☐Unknown;
If done: results ☐Normal ☐Abnormal; **If abnormal:** FVC____ mL, FEV1_____mL;

Respiratory rate: [ ][ ][ ] breaths/minute; **SPO_2**: [ ][ ] % ☐Unknown;

Other tests performed: ☐Done ☐Not done ☐Unknown;
If done: Name of the test ___________________ **Results:** ☐Normal ☐Abnormal

Was a gastrointestinal test done? ☐Yes ☐No ☐Unknown;
If yes, specify type of test and results below:

Dysphagia Severity Scale: ☐Done ☐Not done ☐Unknown;
If done: ☐Score 1 ☐Score 2 ☐Score 3 ☐Score 4 ☐Score 5 ☐Score 6 ☐Score 7 ☐Unknown;

Other tests performed: ☐Done ☐Not done ☐Unknown;
If done: Name of the test ___________________ **Results:** ☐Normal ☐Abnormal

Was a musculoskeletal test done? ☐Yes ☐No ☐Unknown;
If yes, specify type of test and results from list below:

Hand grip strength: ☐Done ☐Not done ☐Unknown;
If done: [ ][ ][ ] Newton OR [ ][ ][ ] /Kg;

MRC Sum Score: ☐Done ☐Not done ☐Unknown;
If done: score between 0-60 [ ][ ];

Timed up and go: ☐Done ☐Not done ☐Unknown;
If done: time taken [ ][ ] seconds;

Other tests performed: ☐Done ☐Not done ☐Unknown;
If done: Name of the test __________________ **Results:** ☐Normal ☐Abnormal

Was any test done for fatigue/pain/activities of daily living? ☐Yes ☐No ☐Unknown;
If yes, specify type of test and results from list below:

Barthel Index Score: ☐Done ☐Not done ☐Unknown;
If done: score between 0-100 [ ][ ][ ];

EQ5D-5L: ☐Done ☐Not done ☐Unknown;
If done: score between 11111-55555 [ ][ ][ ] [ ][ ];

Fatigue Numerical Rating Scale: ☐Done ☐Not done ☐Unknown;
If done: score between 0-10 [ ][ ];

Fatigue Severity Scale: ☐Done ☐Not done ☐Unknown;
If done: score between 1-7 [ ][ ];

Pain Numerical Rating Scale: ☐Done ☐Not done ☐Unknown;
If done: score between 0-10 [ ][ ];

Other tests performed: ☐Done ☐Not done ☐Unknown;
If done: Name of the test _____________________ **Results:** ☐Normal ☐Abnormal

World Health Organization

3.4 Clinical Tests and Scales *continuation*
**Was a mental health test done?** Yes No Unknown;
**If yes**, specify type of test and results below:<br>**Hospital Anxiety and Depression Scale**: Done Not done Unknown;<br>**If done:** score between 0-21 [ ][ ];<br>**Impact of Event Scale-Revised**: Done Not done Unknown;<br>**If done:** score between 0-88 [ ][ ];<br>**Patient Health Questionnaire-9 for depression** (PHQ-9 for depression): Done Not done Unknown;<br>**If done:** score between 0-27 [ ][ ];<br>**PTSD Checklist-5**: Done Not done Unknown;<br>**If done:** score between 0-80 [ ][ ];<br>**Other tests performed:** Done Not done Unknown;<br>**If done:** Name of the test ____________________ **Results:** Normal Abnormal
**Other test performed**: Done Not done Unknown;<br>**If done:** Name of the test _____________________ **Results:** Normal Abnormal

3.5 New diagnosis of illness or complication related to COVID-19	
Was the participant newly diagnosed with any illness or complication related to COVID-19 during this visit?	
**Cardiovascular:** Yes No Unknown; **If yes**, please specify diagnosis from the list below:	
**Acute heart failure:**	Yes No Unknown;
**Atrial arrhythmia:**	Yes No Unknown;
**Arterial thrombosis:**	Yes No Unknown;
**Chronic heart failure:**	Yes No Unknown;
**Coronary aneurysms:**	Yes No Unknown;
**Deep vein thrombosis:**	Yes No Unknown;
**Deterioration of prior chronic heart failure:**	Yes No Unknown;
**Ischemic cardiomyopathy:**	Yes No Unknown;
**Left ventricular diastolic dysfunction:**	Yes No Unknown;
**Myocarditis:**	Yes No Unknown;
**Pericarditis:**	Yes No Unknown
**Right ventricular dysfunction:**	Yes No Unknown;
**Ventricular arrhythmia:**	Yes No Unknown
**Other cardiovascular:**	Yes No Unknown; if Yes, specify______________________
**Dermatological:** Yes No Unknown; **If yes**, please specify diagnosis from the list below:	
**COVID toes** (lumpy lesions on toes):	Yes No Unknown;
**Skin rash:**	Yes No Unknown;
**Other dermatological:**	Yes No Unknown; if Yes, specify______________________
**Endocrine:** Yes No Unknown; **If yes**, please specify diagnosis from the list below:	
**Hypothyroidism:**	Yes No Unknown;
**Low insulin sensitivity:**	Yes No Unknown;
**Thyroiditis:**	Yes No Unknown;
**Other endocrine:**	Yes No Unknown; if Yes, specify______________________
**Gastro-intestinal***:* Yes No Unknown; **If yes,** please specify diagnosis from the list below:	
**Deterioration of prior chronic liver failure:**	Yes No Unknown;
**Dysphagia:**	Yes No Unknown;
**Gastrointestinal haemorrhage**:	Yes No Unknown;
**Post-infectious Irritable Bowel Syndrome**:	Yes No Unknown;
**Other gastrointestinal:**	Yes No Unknown; if Yes, specify______________________
**Generic:** Yes No Unknown; **If yes**, please specify diagnosis from the list below:	
**Exertional fatigue:**	Yes No Unknown;
**Post viral fatigue syndrome:**	Yes No Unknown;
**Other generic:**	Yes No Unknown; if Yes, specify______________________

World Health Organization

**3.5 New diagnosis of illness or complication related to COVID-19** *continuation*	
**Musculoskeletal:** ☐Yes ☐No ☐Unknown; **If yes**, please specify diagnosis from the list below:	
**Arthralgia:**	☐Yes ☐No ☐Unknown;
**Arthritis:**	☐Yes ☐No ☐Unknown;
**ICU acquired weakness:**	☐Yes ☐No ☐Unknown;
**Myalgia:**	☐Yes ☐No ☐Unknown;
**Myositis:**	☐Yes ☐No ☐Unknown;
**Muscle atrophy:**	☐Yes ☐No ☐Unknown;
**Muscle weakness:**	☐Yes ☐No ☐Unknown;
**Osteopenia:**	☐Yes ☐No ☐Unknown;
**Osteoporosis:**	☐Yes ☐No ☐Unknown;
**Secondary sarcopenia:**	☐Yes ☐No ☐Unknown;
**Other musculoskeletal**:	☐Yes ☐No ☐Unknown; if Yes, specify______________________
**Mental health:** ☐Yes ☐No ☐Unknown; **If yes**, please specify diagnosis from the list below:	
**Anxiety:**	☐Yes ☐No ☐Unknown;
**Depression:**	☐Yes ☐No ☐Unknown;
**Post-traumatic Stress Disorder:**	☐Yes ☐No ☐Unknown;
**Psychosis:**	☐Yes ☐No ☐Unknown;
**Sleep disorder:**	☐Yes ☐No ☐Unknown;
**Other mental:**	☐Yes ☐No ☐Unknown; if Yes, specify______________________
**Neurological:** ☐Yes ☐No ☐Unknown; **If yes,** please specify diagnosis from the list below:	
**Demyelinating or other inflammatory white matter brain lesions**: ☐Yes ☐No ☐Unknown;	
**Dementia/other neurocognitive disorder:**	☐Yes ☐No ☐Unknown;
**Dysautonomia:**	☐Yes ☐No ☐Unknown;
**Encephalitis:**	☐Yes ☐No ☐Unknown;
**Headache:**	☐Yes ☐No ☐Unknown;
**Hearing impairment:**	☐Yes ☐No ☐Unknown;
**Hemorrhagic Stroke:**	☐Yes ☐No ☐Unknown;
**Hypoxic ischemic brain injury**:	☐Yes ☐No ☐Unknown;
**Intracerebral haemorrhage:**	☐Yes ☐No ☐Unknown;
**Intraventricular haemorrhage:**	☐Yes ☐No ☐Unknown;
**Ischemic Stroke:**	☐Yes ☐No ☐Unknown;
**Meningitis:**	☐Yes ☐No ☐Unknown;
**Movement Disorder:**	☐Yes ☐No ☐Unknown;
**Motor Neuron Disease:**	☐Yes ☐No ☐Unknown;
**Myelopathy/Spinal Cord Disease**:	☐Yes ☐No ☐Unknown;
**Myopathy:**	☐Yes ☐No ☐Unknown;
**Neuromuscular Disorders:**	☐Yes ☐No ☐Unknown;
**Neuromuscular junction disorder:**	☐Yes ☐No ☐Unknown;
**Non-traumatic subarachnoid haemorrhage:**	☐Yes ☐No ☐Unknown;
**Polyneuropathy:**	☐Yes ☐No ☐Unknown;
**Polyradiculoneuropathy (GBS):**	☐Yes ☐No ☐Unknown;
**Psychiatric disorder:**	☐Yes ☐No ☐Unknown;
**Plexopathy:**	☐Yes ☐No ☐Unknown;
**Radiculopathy:**	☐Yes ☐No ☐Unknow;
**Seizures/Epilepsy:**	☐Yes ☐No ☐Unknown;
**Toxic/Metabolic Encephalopathy:**	☐Yes ☐No ☐Unknown;
**Vision impairment:**	☐Yes ☐No ☐Unknown;
**Other neurological:**	☐Yes ☐No ☐Unknown; if Yes, specify______________________
**Pulmonary:** ☐Yes ☐No ☐Unknown; **If yes**, please specify diagnosis from the list below:	
**Bronchiectasis**:	☐Yes ☐No ☐Unknown;
**Cystic changes**:	☐Yes ☐No ☐Unknown;
**Deterioration of prior chronic pulmonary disease**: ☐Yes ☐No ☐Unknown;	
**Lung fibrosis**:	☐Yes ☐No ☐Unknown;
**Lung hypoperfusion**:	☐Yes ☐No ☐Unknown;
**Mixed restrictive and obstructive pulmonary disease:** ☐Yes ☐No ☐Unknown;	
**Obstructive pulmonary disease:**	☐Yes ☐No ☐Unknown;
**Pleural lesions:**	☐Yes ☐No ☐Unknown;
**Pulmonary arterial hypertension:**	☐Yes ☐No ☐Unknown;
**Pulmonary embolism**:	☐Yes ☐No ☐Unknown;
**Restrictive pulmonary disease:**	☐Yes ☐No ☐Unknown;
**Other pulmonary:**	☐Yes ☐No ☐Unknown; if Yes, specify______________________
**Renal:** ☐Yes ☐No ☐Unknown; **If yes**, please specify diagnosis from the list below:	
**Chronic renal failure:**	☐Yes ☐No ☐Unknown;
**Deterioration of prior chronic renal failure:**	☐Yes ☐No ☐Unknown;
**Other renal:**	☐Yes ☐No ☐Unknown; if Yes, specify______________________
